# Quantifying Individual Response to PRRSV Using Dynamic Indicators of Resilience Based on Activity

**DOI:** 10.3389/fvets.2020.00325

**Published:** 2020-06-24

**Authors:** Lisette E. van der Zande, Jenelle R. Dunkelberger, T. Bas Rodenburg, J. Elizabeth Bolhuis, Pramod K. Mathur, W. James Cairns, Michael C. Keyes, John M. Eggert, Erin A. Little, Scott A. Dee, Egbert F. Knol

**Affiliations:** ^1^Adaptation Physiology Group, Wageningen University & Research, Wageningen, Netherlands; ^2^Topigs Norsvin USA, Burnsville, MN, United States; ^3^Animals in Science and Society, Faculty of Veterinary Medicine, Utrecht University, Utrecht, Netherlands; ^4^Topigs Norsvin Research Center, Beuningen, Netherlands; ^5^Remote Insights, Minneapolis, MN, United States; ^6^Pipestone Applied Research, Pipestone, MN, United States

**Keywords:** resilience, accelerometer, dynamic indicator of resilience, activity, pig behavior

## Abstract

Pigs are faced with various perturbations throughout their lives, some of which are induced by management practices, others by natural causes. Resilience is described as the ability to recover from or cope with a perturbation. Using these data, activity patterns of an individual, as well as deviations from these patterns, can potentially be used to quantify resilience. Dynamic indicators of resilience (DIORs) may measure resilience on a different dimension by calculating variation, autocorrelation and skewness of activity from the absolute activity data. The aim of this study was to investigate the potential of using DIORs of activity, such as average, root mean square error (RMSE), autocorrelation or skewness as indicators of resilience to infection with the Porcine Reproductive and Respiratory Syndrome Virus (PRRSV). For this study, individual activity was obtained from 232 pigs equipped with ear tag accelerometers and inoculated with PRRSV between seven and 9 weeks of age. Clinical scores were assigned to each individual at 13 days post-challenge and used to distinguish between a resilient and non-resilient group. Mortality post-challenge was also recorded. Average, RMSE, autocorrelation and skewness of activity were calculated for the pre- and post-challenge phases, as well as the change in activity level pre- vs. post-challenge (i.e., delta). DIORs pre-challenge were expected to predict resilience to PRRSV in the absence of PRRSV infection, whereas DIORs post-challenge and delta were expected to reflect the effect of the PRRSV challenge. None of the pre-challenge DIORs predicted morbidity or mortality post-challenge. However, a higher RMSE in the 3 days post-challenge and larger change in level and RMSE of activity from pre- to post-challenge tended to increase the probability of clinical signs at day 13 post-infection (poor resilience). A higher skewness post-challenge (tendency) and a larger change in skewness from pre- to post-challenge increased the probability of mortality. A decrease in skewness post-challenge lowered the risk of mortality. The post-challenge DIOR autocorrelation was neither linked to morbidity nor to mortality. In conclusion, results from this study showed that post-challenge DIORs of activity can be used to quantify resilience to PRRSV challenge.

## Introduction

Resilience is defined as the ability to rapidly recover from or cope with a perturbation (1). Perturbations can be of any natural cause (e.g., heat stress) or can, in the case of farm animals, be induced by management practices (e.g., transportation). Pigs face multiple perturbations during their lives. When exposed to a perturbation, pigs may show individual differences in resilience. Improving resilience in pigs may contribute to sustainable pig production for a number of reasons. Resilient pigs are better able to recover from perturbations, including infectious challenges, and require fewer treatments and management interventions. The improved overall health status of resilient animals also result in improved animal welfare. In addition, because resilient pigs are less disturbed by a perturbation, they require less feed than non-resilient pigs for the same amount of growth, and therefore have a better feed efficiency (2). For these reasons, promoting resilience in pigs by optimizing (early life) conditions or by genetic selection, is desirable for future pig production.

Resilience may be measured in various ways, for instance by using physiological parameters. Blood parameters, such as white blood cell count and hemoglobin level, are examples of physiological parameters used as indicators of resilience (3). Other physiological variables used are production parameters like body weight and milk yield, which are commonly used to predict health related traits (4, 5). However, despite the number of parameters used, the lack of a golden standard for quantifying resilience remains a challenge. Assessment of physiological parameters can be invasive to the animals, and is often labor intensive. Moreover, it is often not feasible to collect physiological data repeatedly, whereas for assessment of recovery time following a perturbation, frequent, or continuous measurements are required. Behavior is one example of a non-invasive parameter with the potential for easy, repeatable observations. Weary et al. (6) stated that behavior is the most commonly used indicator for illness, as reduced activity is a main characteristic of the sickness response that is induced after infection (7), and may also occur after other stressors (8). Locomotor behavior is therefore often included in the ethogram of studies investigating illness. Traditional behavioral observation methods are labor intensive, especially when animals need to be studied frequently. Precision phenotyping tools, such as wearable accelerometers, which are capable of quantifying activity automatically, are therefore an attractive alternative. Accelerometers measure acceleration along the *x, y*, and *z*-axis. Using machine learning models, acceleration can be translated to activity which can, in turn, possibly be used to quantify resilience.

Apart from changes in the level of activity *per se*, dynamic changes in activity patterns may be related to resilience (9). Dynamic indicators of resilience (DIORs), which are capable of quantifying deviations in functioning of biological systems, are proposed by Scheffer et al. (10) and have been adopted for farm animals as resilience indicators (4). Such DIORs are, for instance, variance, and autocorrelation in repeatedly measured variables, which may include activity. It is expected that resilient pigs will show less variation in activity following a perturbation. In general, the activity level of pigs following a health challenge will be reduced. Pigs that recover more quickly from such a challenge (i.e., resilient pigs) will return to their initial level of activity faster than non-resilient pigs. This should result in a lower Root Mean Square Error (RMSE) of activity. Putz et al. (11) found a positive genetic correlation between RMSE of feed intake and mortality, suggesting that RMSE of feed intake can be used as an indicator of resilience. Autocorrelation represents the degree of similarity between two given time periods and ranges from −1 to 1. It is hypothesized that resilient pigs will have a (lag-1) autocorrelation of activity around zero (12), as their fast recovery results in less resemblance to previous days. Less resilient pigs recover more slowly from a perturbation, resulting in more similarity in activity of previous days for a longer period of time, i.e., a high autocorrelation. Skewness indicates the direction of the response to perturbation, i.e., a positive or negative response. It is expected that resilient pigs will have a skewness around zero as they recover more quickly from a perturbation than non-resilient pigs. All DIORs are expected to be most informative immediately following a perturbation. It can be observed directly whether a decrease in activity occurs, how steep the slope of the decrease is, and how long it persists. However, it has been suggested that dynamic patterns in repeatedly measured biological systems before a major perturbation might also be predictive of resilience. Systems losing resilience, approaching a tipping point to an alternative state (e.g., disease) may also show slower recovery from small, natural perturbations in the environment, resulting in, for instance, higher autocorrelation, and variance [see (10), for review].

In this study, DIORs based on activity were used to measure and potentially predict resilience following a Porcine Reproductive and Respiratory Syndrome Virus (PRRSV) infection. PRRSV is a common infection among pig populations (13). As its name implies, PRRSV results in two main pathologies: reproductive failure and respiratory disease. Reproductive failure occurs in pregnant sows and results in abortions, mummified piglets, and weak live born piglets. Growing pigs infected with PRRSV may suffer from high fever, have loss of appetite and become lethargic or less active, leading to reduced growth and feeding efficiency, and increased mortality. The course of the clinical signs is on average 2 weeks. Despite the availability of vaccines, PRRS remains a difficult disease to control and regular outbreaks occur. Besides the impairment of pig welfare, PRRSV causes severe economic losses for the farmer.

The aim of this study was to investigate whether activity levels, or DIORs such as RMSE, autocorrelation or skewness of activity patterns, can be used as dynamic indicators of resilience following PRRSV infection in pigs.

## Materials and Methods

Data for this paper were obtained from a subset of pigs in an experiment executed by Pipestone Veterinary Research and Topigs Norsvin USA. Prior to the start of that experiment, Pipestone Applied Research (PAR) institutional animal care and use committees (PAR IACUC 1-18) reviewed and approved the trial.

### Animals and Housing

A total of 2,186 commercial crossbred pigs from a commercial sow farm were used for the study we obtained data from. Upon weaning at approximately 3 weeks of age, pigs were shipped to a commercial research facility in the US. Each pen had fully slatted floors, with 2 cup waterers and a 4-hole dry feeder which provided 35 cm of feeder space per pig. Feed and water were provided *ad libitum*. Pigs originated from three genetic groups. Two groups were sired by boars from the same genetic line, but these boars were selected based on a different breeding goal. The third group was sired by a different genetic line. Upon arrival at the research facility, pigs were penned by genetic group and balanced by sex with 27 pigs housed per pen (0.65 m^2^/pig) in 81 pens in total and all pigs were vaccinated per the label instructions using a PRRS modified live virus vaccine (IngelVac ATP, Boehringer Ingelheim). Pens had fully slatted concrete floors. Lights were on in the facility from 8:00 to 20:00 with a night light turned on outside of these hours. Four weeks later, pigs were experimentally inoculated with PRRS virus variant 1-7-4 at a total dose of 1 × 10^5^ TCID50 via the IM route [SD15-174 (lineage 1)-TB3-P8, SDSU, Brookings, USA] (14). At 0, 13, and 42 days post-infection, corresponding with expected peak PRRS viremia and viral clearance at 13 and 42 days post-infection, pigs were weighted and clinical scores were assigned using a 6-point scoring system (15, 16). Scores were assigned as follows where: “1,” healthy; “2,” mild signs of disease; “3,” moderate signs of disease; “4,” advanced signs of disease; “5,” extreme signs of disease; and “6,” deceased (including day) (17). We could not define the recovery period using activity, because clinical scores were not assessed daily. Therefore, clinical scores at 13 days post-infection were used to distinguish pigs with a favorable or unfavorable outcome of the infection, where pigs with a clinical score of “1” were classified as “resilient,” and pigs with a clinical score “>1” were classified as “non-resilient.”

### Collection of Accelerometer Data

A subset of 232 pigs, originating from 9 pens (3 pens per genetic group), were equipped with individual accelerometer ear tags at 5 weeks of age (Remote Insights, Minneapolis, USA). Accelerometer data were recorded from 23 days prior to infection with PRRSV to 42 days post-infection. Videos of the pigs were annotated for activity by Remote Insights. The annotations were used as training and validation data for a machine learning model to classify their activity (Remote Insights, Minneapolis, USA). A 5-s window was classified as active or inactive, based on the output of the machine learning model, which resulted in 720 windows per hour. Data were transformed to minutes per hour. Forty-seven animals were removed from the final dataset, due to missing data for more than 20 consecutive hours, resulting in a total of 185 animals used for analyses. Missing values influence the calculation of DIORs. To avoid this, a rolling average was used for the analysis with a window of 12 h.

### DIORs Calculation

Dynamic indicators of resilience (DIORs) were calculated per individual for the pre-challenge (from 23 days pre-challenge until challenge) and post-challenge (from challenge until 3 days post-challenge) phases, as well as the change in activity level from 3 days pre-challenge vs. 3 days post-challenge (i.e., delta). Pre-challenge data were used to potentially predict resilience, based on clinical scores on day 13 post-challenge, without the influence of the PRRSV inoculation. DIORs post-challenge, based on data from the first 3 days post-challenge, were also used to potentially predict resilience and mortality. The first 3 days post-challenge were chosen, because on the fourth day post-challenge the first pig died, so all animals have data collection up to 3 days post-challenge. The delta of DIORs following inoculation was calculated by subtracting DIORs of 3 days pre-challenge from DIORs of 3 days post-challenge.

Root Mean Square Error (RMSE) of activity of the *j*^th^ individual was calculated as:

$$\begin{array}{l} RMSE_{j}=\sqrt{\frac{\sum_{i=1}^{n_{j}}{\left(x_{f_{ij}}-x_{o_{ij}}\right)}^{2}}{n_{j}}}, \end{array}$$

where *x*_*f*_*ij*__ is the forecasted observation *i* of the *j*^th^ individual, *x*_*o*_*ij*__ is the observed observation *i* of the *j*^th^ individual, and *n*_*j*_ is the number of observations of the *j*^*th*^ individual.

Autocorrelation of activity of the *j*^th^ individual was calculated as:

$$\begin{array}{l} Autocorrelation_{j}=\frac{\sum_{i=1}^{n_{j}-k}\left(x_{ij}-{\bar{x}}_{j})(x_{(i+k)j}-{\bar{x}}_{j}\right)}{\sum_{i=1}^{n_{j}}{\left(x_{ij}-\;{\bar{x}}_{j}\right)}^{2}}, \end{array}$$

Where *n*_*j*_ is the number of observations of the *j*^th^ individual, *x*_*ij*_ the *i*^th^ observation of the *j*^th^ individual, and ${\bar{x}}_{j}$ the sample mean of the *j*^th^ individual.

Skewness of activity of the *j*^th^ individual was calculated as:

$$\begin{array}{l} Skewness_{j}=\frac{\sqrt{n_{j}\left(n_{j}-1\right)}}{n_{j}-2}\;\frac{m_{3}}{m_{2}^{3/2}}, \end{array}$$

where *n*_*j*_ is the number of observations of the *j*^th^ individual, $m_{k}=\frac{1}{n_{j}}\overset{n_{j}}{\underset{i\;=\;1}{\sum }}{\left(x_{ij}-{\bar{x}}_{j}\right)}^{k}\;$, where *x*_*ij*_ is the *i*^th^ observation of the *j*^th^ individual, and ${\bar{x}}_{j}$ the sample mean of the *j*^th^ individual.

### Statistical Analysis

All models were fitted using R (18). A generalized linear mixed model using a binomial distribution with logit link function was used to test whether DIORs were different for resilient and non-resilient pigs (based on assigned clinical scores). DIORs were tested independent of each other. Fixed effects in the generalized linear mixed model were DIOR and clinical score at the day of inoculation as some pigs already had early or moderate signs of clinical disease. Pen was included as a random effect. Mortality was tested using Cox regression survival analysis. Fixed effects in the Cox regression model were DIOR and clinical score at the day of inoculation. Pen was included as a random effect.

## Results

Two pigs had died prior to inoculation. At day 13 post-challenge, 92 pigs had a clinical score of “1” (i.e., resilient group), where 93 pigs had a clinical score of “2” or greater (i.e., non-resilient group). The resilient group had significantly (*P* < 0.001) higher average daily gain between inoculation and day 13 post-challenge compared to the non-resilient group (0.47 ± 0.02 vs. 0.23 ± 0.02 kg). At day 13 post-challenge, 7 pigs had died between 1 day pre-challenge and 12 days post-challenge. By the end of the study (at 42 days post-challenge), 13 pigs had died between 1 day pre-challenge, and 27 days post-challenge. Table 1 shows the means and standard deviations of DIORs pre- and post-challenge, illustrating that the average activity levels decreased following challenge, whereas the impact on other DIORs was minimal.

**Table 1:** Means and corresponding standard deviation in parentheses for DIORs of activity (min/hour) pre-challenge and post-challenge.

**DIOR**	**Pre-challenge^**a**^**	**Post-challenge^**b**^**
Average activity^c^	12.17 (1.63)	8.41 (2.00)
RMSE of activity^c^	3.75 (0.60)	3.60 (0.97)
Autocorrelation of activity	0.94 (0.01)	0.91 (0.03)
Skewness of activity	0.24 (0.34)	0.31 (0.38)

a *Pre-challenge is from 23 days pre-challenge until challenge*.

b Post-challenge is from challenge until 3 days post-challenge.

c *In minutes per hour*.

### Association Between DIORs Pre-challenge and Morbidity and Mortality

Odds ratios given in Tables 2, 4 reflect the probability of being non-resilient, i.e., showing clinical signs at day 13 post infection, over the probability of being resilient. The hazard ratios presented in Tables 3, 5 give the probability of mortality in respect of time.

**Table 2:** Odds ratios with 95% confidence intervals (CI) for DIORs of activity pre-challenge (based on 23 days) using generalized linear mixed models for resilience (i.e. morbidity) following PRRSV inoculation.

**DIOR^**a**^**	**Odds ratio (95% CI)**	***P*-value**
Average activity	1.14 (0.92–1.40)	0.32
RMSE of activity	1.14 (0.66–1.97)	0.61
Skewness of activity	0.99 (0.36–2.77)	0.71

a *Odds ratio of autocorrelation could not be estimated. The variation in autocorrelation was minimal, resulting in very high confidence intervals*.

**Table 3:** Hazard ratios with 95% confidence intervals (CI) for DIORs of activity pre-challenge (based on 23 days) using Cox regression models for mortality following PRRSV inoculation.

**DIOR^**a**^**	**Hazard ratio (95% CI)**	***P*-value**
Average activity	1.10 (0.77–1.60)	0.60
RMSE of activity	1.24 (0.49–3.20)	0.65
Skewness of activity	0.27 (0.04–1.40)	0.11

a *Hazard ratio of autocorrelation could not be estimated. The variation in autocorrelation was minimal, resulting in very high confidence intervals*.

**Table 4:** Odds ratios with 95% confidence intervals (CI) of DIORs of activity 3 days post-challenge using generalized linear mixed model for resilience (i.e. morbidity) following PRRSV inoculation.

**DIOR^**a**^**	**Odds ratio (95% CI)**	***P*-value**
Average activity	1.04 (0.88–1.24)	0.65
RMSE of activity	1.42 (1.01–2.05)	0.05
Skewness of activity	1.30 (0.56–3.04)	0.54

a *Odds ratio of autocorrelation could not be estimated. The variation in autocorrelation was minimal, resulting in very high confidence intervals*.

**Table 5:** Hazard ratios with 95% confidence intervals (CI) of DIORs of activity 3 days post-challenge using Cox regression models for mortality following PRRSV inoculation.

**DIOR^**a**^**	**Hazard ratio (95% CI)**	***P*-value**
Average activity	0.80 (0.58–1.10)	0.18
RMSE of activity	1.09 (0.59–2.00)	0.78
Skewness of activity	3.02 (0.92–10.00)	0.07

a *Hazard ratio of autocorrelation could not be estimated. The variation in autocorrelation was minimal, resulting in very high confidence intervals*.

DIORs pre-challenge did not relate to the probability of being non-resilient (Table 2). In addition, probability of mortality post-challenge could not be predicted by DIORs pre-challenge (Table 3).

### Association Between DIORs of Activity Post-challenge and Morbidity and Mortality

RMSE of activity 3 days post-challenge tended to be different between resilient and non-resilient groups (Table 4). The odds ratio of RMSE indicates that for every one-unit increase in RMSE, the odds of being non-resilient increases by 1.42 times. Skewness of activity tended to relate to mortality (Table 5). Every one-unit increase in skewness, the relative risk of mortality tended to increase 3.02 times.

### Association Between Change in DIORs From Pre- to Post-challenge and Morbidity and Mortality

The change in DIORs was calculated by subtracting the DIOR for 3 days pre-challenge from the DIOR for 3 days post-challenge. Table 6 shows that changes in average activity and RMSE from pre-challenge to post-challenge tended to affect the probability of a non-resilient outcome of the infection. When the average activity decreased post-challenge by one-unit, the probability of being non-resilient was 22% higher (1 divided by 0.82). The effect of changes in RMSE was in the opposite direction. One-unit increase in RMSE tended to increase the odds of being non-resilient by 1.34. The change in skewness significantly affected the probability of mortality (Table 7). For every one-unit increase in skewness, the relative risk of mortality increased by 3.70.

**Table 6:** Odds ratios with 95% confidence intervals (CI) of the difference in DIORs of activity pre-challenge and post-challenge using generalized linear mixed models (*n* = 185) for resilience (i.e. morbidity) groups following PRRSV inoculation.

**DIOR^**a**^**	**Odds ratio (95% CI)**	***P*-value**
Average activity	0.82 (0.66–1.01)	0.06
RMSE of activity	1.34 (0.98–1.87)	0.07
Skewness of activity	1.18 (0.56–2.22)	0.75

a *Odds ratio of autocorrelation could not be estimated. The variation in autocorrelation was minimal, resulting in very high confidence intervals*.

**Table 7:** Hazard ratios with 95% confidence intervals (CI) of the difference in DIORs of activity pre-challenge and post-challenge using Cox regression models for mortality following PRRSV inoculation.

**DIOR^**a**^**	**Hazard ratio (95% CI)**	***P*-value**
Average activity	0.79 (0.52–1.20)	0.23
RMSE of activity	1.21 (0.66–2.20)	0.54
Skewness of activity	3.70 (1.5–9.0)	0.004

a *Hazard ratio of autocorrelation could not be estimated. The variation in autocorrelation was minimal, resulting in very high confidence intervals*.

## Discussion

This study investigated the use of DIORs, including average, RMSE, autocorrelation, and skewness of activity to quantify resilience following PRRSV infection. It was expected that DIORs pre-challenge could be predictive of morbidity or mortality post-challenge. However, no DIOR pre-challenge was identified as predictive for morbidity or mortality in this study. Previous studies that investigated DIORs in livestock calculated DIORs using the entire study period, including the challenge period. This study identified associations between DIORs based on activity and resilience after the PRRSV challenge only, indicating that these DIORs are only associated with resilience when the animal is challenged.

To our knowledge, this is the first study to investigate pre-challenge DIORs as potential indicators of resilience in livestock. Gijzel et al. (19) explored the association between DIORs and frailty levels of elderly people. Results showed greater variation in the physical, mental, and social domain, for frail elderly individuals than non-frail elderly individuals. It should be noted, though, that in this between-subject study within-subject changes in resilience were not investigated. Thus, although DIORs pre-challenge may be associated with resilience, results from this study did not support predictive value of DIORs related to activity for the recovery of pigs from a PRRVS infection.

It was expected that activity would decrease following PRRSV inoculation, given that sickness behavior is typically characterized by a decrease in locomotor activity (20). The results from this study support this by showing that a decrease in activity post-challenge as compared with pre-challenge levels, increased the risk of being classified as non-resilient, i.e., showing clinical signs on day 13 post challenge. This suggests that changes in activity levels in the early stage of infection may be a useful DIOR following PRRSV infection. Several studies have reported a decrease in activity following PRRSV-infection (7, 21) or other diseases (22). However, occasionally, an increase in activity may be observed post-infection. For example, pigs infected with *Salmonella* were more active (23). Another perturbation, such as regrouping, is also associated with an increase in activity. After regrouping, pigs show an increase in activity (24). Therefore, the desired direction of activity changes for identifying resilient pigs may differ depending on the specific perturbation.

RMSE post-challenge and the change in RMSE following PRRSV inoculation were linked to morbidity. A higher increase in RMSE following and a higher RMSE post-challenge tended to increase the risk of a non-resilient outcome, i.e., morbidity or mortality. No associations were identified between RMSE and mortality alone, whereas Putz et al. (11) found that a higher RMSE of feed intake following natural disease challenge was associated with higher mortality. One possible explanation for this finding could be that a much lower mortality rate was observed for this study (7%) compared to the mortality rate observed by Putz et al. (11) (26%). The perturbation used by Putz et al. (11) included various viral and bacterial diseases, whereas this study used only one experimentally induced viral disease as a perturbation. Furthermore, deviations in feed intake may be more informative for mortality than deviations in activity. Another explanation could be the smaller sample size in this study.

Autocorrelation was expected to be around zero for resilient animals. However, autocorrelation had little to no variation between animals. The confidence interval of odds and hazard ratio had a range of more than one thousand (data not shown). Multiplying autocorrelation by 100 lowered the confidence interval. However, autocorrelation in activity remained uninformative regarding morbidity or mortality. Apart from the possibility that the time series resolution and length may not have been optimal for calculation of this DIOR, not all variables are characterized by critical slowing down, of which autocorrelation is a typical indicator. It has been argued that only time series of physiological variables that are maintained close to a pre-determined setpoint and fluctuate around an equilibrium, “regulated variables” exhibit critical slowing down when resilience is reduced (25). In line with this, Berghof et al. (4) and Poppe et al. (5) concluded that autocorrelation in body weight of layer chickens and milk yield of dairy cattle seem to be less informative for quantifying resilience.

In contrast with RMSE of activity, which tended to be related to morbidity, skewness in activity post-challenge, and particularly the change in skewness from pre- to post-challenge, was associated with mortality rather than morbidity. Skewness was expected to be around zero for resilient animals. Lower skewness post-challenge indeed increased the odds of being resilient. Skewness post-challenge had a mean of 0.31 (Table 1), so a decrease in skewness indicates a movement toward zero. However, skewness has a range of −1 to 1, so a one-unit shift in skewness is very unlikely. Berghof et al. (4) and Poppe et al. (5) concluded that skewness in body weight of layer chickens and milk yield was less informative for health and longevity traits than other DIORs. This is also in line with the findings from this study, which indicate that skewness is not related to morbidity. Skewness could be sensitive to outliers, which could be the case for individual recordings of milk yield and activity (5). Results from this study did, however, identify an association between reduced skewness (movement toward zero) with decreased risk of mortality.

For young animals, activity decreases over time irrespective of a perturbation (26). This study did not correct for this decrease in activity. DIORs post-challenge and their deviations from pre-challenge values were calculated based on 3 days, and it is therefore assumed that the changes in these 3 days are due to the perturbation. To use activity of the whole period, control animals should be added to be able to correct for the decrease in activity due to aging.

The results obtained from this study demonstrated the value of DIORs based on activity to quantify resilience to disease challenge in pigs, although studies with larger sample sizes are needed to confirm this. The accelerometers used in this study measured acceleration using three axes and machine learning models to calculate activity, which is a black box approach. Based on accelerations, activity could be assessed, but spatial distribution, specific behaviors (e.g., whether a pig was shaking its head or running around) or social interactions could not be measured. Conversely, computer vision, allowing for immediate identification of a pig in a video and registering of its coordinates, could be used to extract the location and specific behavior of the animal. Additional information captured using computer vision might include distance moved, velocity, spatial distribution, and social interactions. Taken together, these parameters would allow for the analysis of more complex activity and behavioral traits. Therefore, data generated via computer vision technology may improve estimation of DIORs, compared to using accelerometer data. However, accelerometers are currently commercially available, while camera technology is not yet ready for implementation at the commercial level. In the future, the cost/benefit of accelerometers vs. cameras will need to be evaluated on a case-by-case basis.

## Conclusion

Results from this study showed that DIORs based on activity pre-challenge could not predict morbidity and mortality following a PRRSV infection. However, RMSE in the 3 days post-challenge and the change in RMSE and average activity from pre-to post-challenge tended to be associated with morbidity 13 days after infection. Skewness post-challenge tended to be associated with mortality, and the change in skewness was significantly related to mortality. Thus, DIORs based on activity showed their value to quantify resilience to a disease challenge. To explore the full potential of DIORs more in depth, more elaborate measurements of behavior are desirable. Computer vision may allow for these in-depth measurements which cannot be assessed using accelerometers.

## Data Availability Statement

The datasets generated for this study are available on request to the corresponding author.

## Ethics Statement

This animal study was reviewed and approved by Pipestone Applied Research (PAR) institutional animal care and use committees (PAR IACUC 1-18).

## Author Contributions

JD, PM, JE, EL, SD, and EK designed the experiment and developed protocols for animal sourcing, management, and phenotype recording. WC and MK employed the ear tag accelerometers and generated the activity dataset. LZ analyzed the data and wrote the manuscript with help of TR, JB, JD, and EK. All authors reviewed and approved the final manuscript.

## Conflict of Interest

JD, PM, JE, and EK were employed by Topigs Norsvin. WC and MK were employed by Remote Insights. EL and SD were employed by Pipestone Applied Research. The remaining authors declare that the research was conducted in the absence of any commercial or financial relationships that could be construed as a potential conflict of interest.
